# Chemoradiotherapy for Small-Cell Prostate Carcinoma: A Case Report

**DOI:** 10.7759/cureus.37287

**Published:** 2023-04-08

**Authors:** Kenji Makita, Yasushi Hamamoto, Kei Nagasaki, Hiromitsu Kanzaki, Katsuyoshi Hashine

**Affiliations:** 1 Department of Radiation Oncology, National Hospital Organization Shikoku Cancer Center, Matsuyama, JPN; 2 Department of Radiology, Ehime University Graduate School of Medicine, Toon, JPN; 3 Department of Radiology, Ehime Prefectural Central Hospital, Matsuyama, JPN; 4 Department of Urology, National Hospital Organization Shikoku Cancer Center, Matsuyama, JPN

**Keywords:** chemotherapy, brachytherapy, local control, radiotherapy, small-cell prostate carcinoma

## Abstract

A 60-year-old male with small-cell prostate carcinoma (SCPC) received external-beam radiotherapy of 60 Gy in 30 fractions and chemotherapy (cisplatin (CDDP) 80 mg/m^2^ + etoposide (VP-16) 100 mg/m^2^, six courses). Although fluorodeoxyglucose positron emission tomography/computed tomography (FDG-PET/CT) showed a complete response, local recurrence occurred in the gross tumor volume after 12 months after the end of chemoradiotherapy. Although the standard treatment for SCPC is not established because SCPC is a rare disease, radiotherapy for SCPC is necessary to study the optimal dose and irradiation area for local control.

## Introduction

Small-cell prostate carcinoma (SCPC) is a rare disease that occurs in approximately 1% of primary malignancies of the prostate. In contrast to typical prostate carcinoma, hormone therapy is often ineffective, and the prognosis is unfavorable. The median survival time from diagnosis is one to two years [1]. Although standard treatment has not been established, chemotherapy-based multidisciplinary treatment is often used similar to the treatment of small-cell lung carcinoma. In cases of localized small-cell lung cancer, radiotherapy at approximately 60 Gy in 30 fractions is often used for local treatment [2,3]. In this report, we described a case of local recurrence within the gross tumor volume (GTV) after chemoradiotherapy.

## Case presentation

A 60-year-old male initially presented to the hospital in February 2008 with a chief complaint of general fatigue. A computed tomography (CT) scan revealed the presence of multiple large lymph nodes. To further investigate the extent of the disease, a fluorodeoxyglucose positron emission tomography/CT (FDG-PET/CT) scan was performed, which revealed the FDG accumulation in the prostate and multiple large lymph nodes was observed (standardized uptake value (SUV) max = 6.22 and 5.23, respectively). Furthermore, laboratory data indicated an elevated prostate-specific antigen (PSA) level of 120.35 ng/mL. Subsequently, a needle biopsy of the prostate was performed, and an adenocarcinoma with a Gleason score of 5 + 5 = 10 was detected. Hormonal therapy with goserelin acetate and bicalutamide was initiated for the treatment of prostate carcinoma (cT4N1M1a in UICC 8th). No surgery or radiotherapy was used as a local treatment. PSA level decreased to below the detectable level (<0.003 ng/mL) by June 2010. However, a follow-up CT scan in May 2011 revealed an increase in the size of the primary tumor. A repeat needle biopsy of the prostate revealed the presence of small-cell carcinoma. The patient was subsequently referred to a radiation oncologist to be treated with chemoradiotherapy.

CT scan obtained at the initial visit to the Department of Radiation Oncology

**Figure 1:**
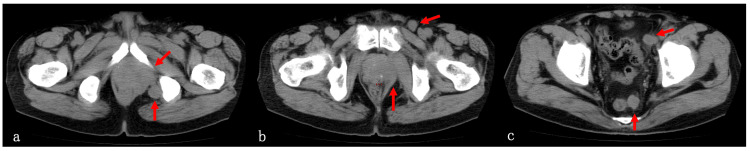
CT image at the initial visit to the Department of Radiation Oncology. (a) Local recurrence, (b) inguinal lymph node metastasis, (c) pelvic lymph node metastasis

The enlarged prostate with a transverse diameter of 62 mm was revealed. The apex of the prostate showed protrusion to the right caudal side, and the prostatic contour was markedly irregular. Local recurrence of prostate carcinoma with extracapsular invasion was suspected. Furthermore, on the dorsal side of the prostate, an enlarged lymph node with a diameter of 2 cm was observed. The right external iliac, inguinal, and presacral lymph nodes were also enlarged. However, no evidence of distant metastasis was detected (Figures 1a-1c).

Histopathologic findings at the initial visit to the Department of Radiation Oncology

**Figure 2:**
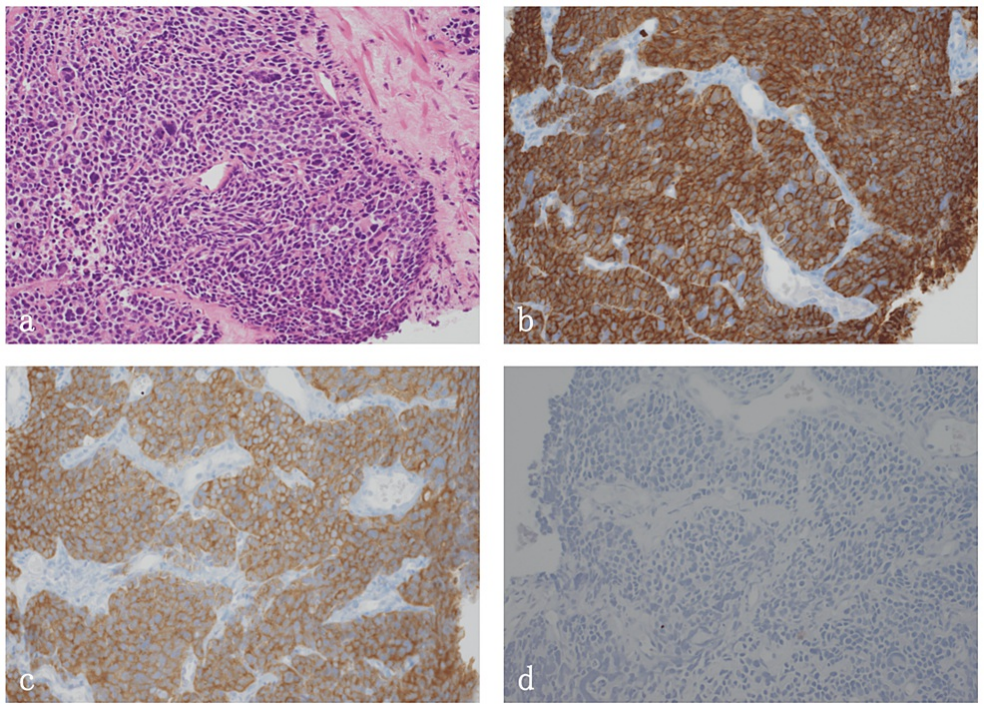
Histological findings. (a) Hematoxylin and eosin staining. (b) CD56 immunostaining. CD56 expression is observed. (c) Synaptophysin immunostaining. Synaptophysin expression is observed. (d) PSA immunostaining. PSA expression is not observed.

In the needle biopsy of the prostate, five out of six samples showed the proliferation of small cells with a high nucleus-to-cytoplasm (N/C) ratio. Although immunostaining for PSA was negative, that for neuroendocrine markers was positive, and high mitotic activity was shown, leading to the diagnosis of prostate small-cell carcinoma (Figures 2a-2d). Immunostaining results showed chromograninA(+), synaptophysin(+), CD56(+), PSA(-), p53/DO7(mutation pattern), and MIB-1 labeling rate of about 80%.

Initial treatment for the SCPC

**Figure 3:**
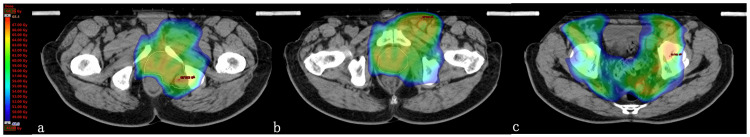
Initial treatment plan of radiotherapy for SCPC. (a) Local recurrence, (b) inguinal lymph node metastasis, (c) pelvic lymph node metastasis. Red line: GTVn, Yellow line: GTVp. Dose-color-wash: 48-68.4 Gy.

External beam radiation therapy (EBRT) with 60 Gy in 30 fractions was administered using a linear accelerator (10 MV x-rays). The prostate and the enlarged pelvic lymph node metastases were included in the GTV, and a 5-mm margin was added in all directions of the GTV to create the clinical target volume1 (CTV1). In addition, a 5-mm margin was added in all directions of the CTV1 to create the planning target volume1 (PTV1). During preparation for intensity-modulated radiation therapy (IMRT), 8 Gy in four fractions was administered to the PTV1 with three-dimensional conformal radiotherapy (3D-CRT) (four fixed beams), and the remaining 52 Gy in 26 fractions was administered with IMRT (seven fixed beams). CTV2 was defined as CTV1 plus pelvic lymph node region below the common iliac artery bifurcation. PTV2 was created by adding a 5-mm margin to CTV2. PTV2 was irradiated 52 Gy in 26 fractions (Dmean prescription) with IMRT (total dose: 60 Gy in 30 fractions, Figures 3a-3c). Etoposide + cisplatin (EP) therapy (cisplatin, 80 mg/m^2^; etoposide, 100 mg/m^2^) was administered concurrently. Because of the possibility of myelosuppression resulting from the combination of radiotherapy and chemotherapy, the chemotherapy was started in the second half of radiotherapy (at 36 Gy in 18 fractions), and a total of six courses of chemotherapy were given.

Progress after initial treatment for SCPC

**Figure 4:**
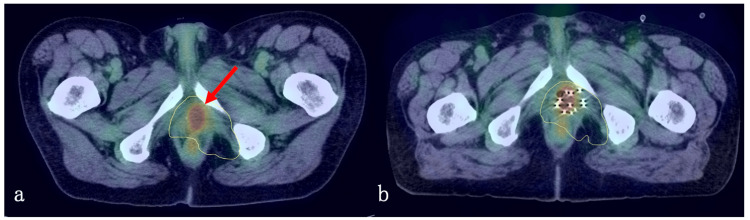
Local recurrence and salvage low-dose-rate brachytherapy. (a) Local recurrence within the GTV, (b) low-dose-rate brachytherapy.

Four months after the start of radiotherapy, a positron emission tomography/CT (PET/CT) scan revealed a decrease in FDG accumulation, and the patient was judged to have a complete response. Thereafter, EP therapy was continued, but one year after the start of EBRT, a PET/CT scan showed a localized FDG accumulation (SUVmax=5.13) within the GTV on the right side of the apex of the prostate, and a local recurrence was suspected (Figure 4a).

Salvage treatment for the local recurrence within the prostate

**Figure 5:**
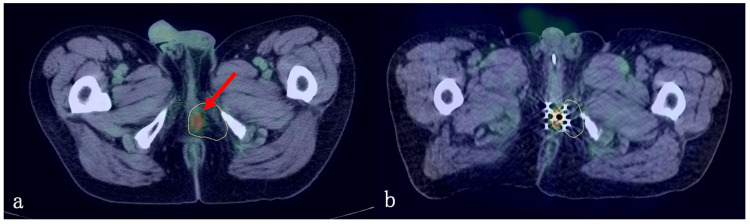
Local recurrence and salvage high-dose-rate brachytherapy. (a) Local recurrence at the lateral margin of the low-dose-rate brachytherapy site, (b) high-dose-rate brachytherapy.

One course of IP therapy (cisplatin, 60 mg/m^2^; irinotecan, 60 mg/m^2^) was administered for local recurrence with the prostate. However, PET/CT scan performed one month later showed a further increase in FDG accumulation (SUVmax=5.99). As salvage therapy to this, low-dose-rate brachytherapy (100 Gy) with 125I seeds was performed on the whole prostate (Figure 4b). The administration of cisplatin was discontinued because of auditory impairment that appeared after the first course of IP therapy. Chemotherapy with irinotecan alone was continued. After six months of 125I brachytherapy, a PET/CT scan revealed a decrease in FDG accumulation (SUVmax=2.90), and the response to the treatment was judged to be a partial response. However, after 13 months of 125I brachytherapy, a mass lesion with increased FDG accumulation (SUVmax=6.88) was diagnosed as a local recurrence at the lateral margin of the 125I brachytherapy site in the perineum area (Figure 5a). High-dose-rate brachytherapy of 19 Gy in two fractions using the 192Ir remote afterloading system (192Ir-RALS) was performed for local recurrence, and 3D-CRT of 20 Gy in 10 fractions was added for the equivalent dose in 2 Gy fraction (α/β = 3) ≥ 60 Gy equivalent (Figure 5b). After these treatments, chemotherapy was terminated. After three months of these treatments, PET/CT scan showed decreased FDG accumulation in the local recurrence (SUVmax=5.1), but a new mass lesion showing increased FDG accumulation (SUVmax=7.8) appeared at the edge of PTV1 (inside PTV2, outside PTV1). The patient underwent high-dose-rate brachytherapy of 19 Gy in two fractions using 192Ir-RALS for this recurrent mass. However, after three months of this treatment, the CT scan showed increased recurrent lesions (1) on the right side of the apex of the prostate (within GTV), (2) on the perineum (field margins of brachytherapy), and (3) on the left dorsal side of the bladder (within PTV2). Since further radiotherapy seemed to be ineffective in controlling the disease, we applied to the institutional ethics board for the use of amrubicin, which is approved for small-cell lung cancer, and started treatment at the patient’s own expense. Amrubicin was effective to keep the disease stable for a while, but disease progression was observed 13 months after the start of Amrubicin. A CT scan revealed disseminated lesions and subcutaneous metastases. Administration of Amrubicin was terminated, and the patient died of prostate carcinoma 43 months after the diagnosis of SCPC. A severe adverse effect attributed to radiotherapy was colostomy necessitated by colorectal damage during the best supportive care phase, 25 days before death.

## Discussion

SCPC is a rare prostate carcinoma with a remarkably poorer prognosis compared to the typical prostate carcinoma. Histologically, SCPC is divided into three types: (1) pure type of small-cell carcinoma, (2) mixed type of adenocarcinoma and small-cell carcinoma, or (3) appearance of small-cell carcinoma during adenocarcinoma treatment. Approximately 50% of SCPC patients are estimated to have a history of adenocarcinoma [4]. The unfavorable prognostic factors for SCPC are advanced stage, mixed type of poorly differentiated adenocarcinoma and small-cell carcinoma, pure type of small-cell carcinoma, or older age [5].

Treatment modalities for SCPC have not yet been established because of their rarity. Generally, chemotherapy is considered the mainstay of multidisciplinary treatment, while radiotherapy is often used as a local treatment in the vigorous proliferation of locoregional lesions. Chemotherapy regimens for SCPC are similar to those for small-cell lung carcinoma [3], with the combination of platinum and etoposide or irinotecan. At our hospital, only these two chemotherapy regimens are registered for small-cell carcinomas other than small-cell lung carcinoma, and third-line chemotherapy is not available. In addition, radiotherapy of 60 Gy in 30 fractions is often administered as local treatment [2,3].

In our case, the initial salvage treatment (EP therapy and EBRT of 60 Gy in 30 fractions) achieved a partial response; however, one year later, recurrence within the GTV (received 60 Gy) was observed. Hashimoto et al. reported an in-field recurrence (within a high-dose irradiation field of 70 Gy in 35 fractions) after IMRT and hormonal therapy, although chemotherapy was not used in their case [6]. Generally, in typical prostate carcinoma, the dose of 74-78 Gy in 37-39 fractions is used depending on the risk classification [3]. Because the dose of 60-70 Gy in 30-35 fractions may be inadequate for the local control of SCPC, higher doses using precise techniques, such as IMRT, may be adequate for administering local treatment to SCPC.

In typical prostate carcinoma, prophylactic irradiation to the pelvic lymph nodes is controversial [7]. Similarly, the usefulness of prophylactic irradiation to the pelvic lymph node in SCPC has not yet been established. In recent years, a trend toward omitting prophylactic irradiation to the mediastinum in localized small-cell lung carcinoma has emerged [8]. In our case, recurrences occurred repeatedly within the GTV or at the GTV margins. Repeated local treatment for local recurrence prevented distant metastasis for three years after the first local recurrence. Although further studies are needed to establish radiotherapy for SCPC, a higher dose may be required to control gross lesions. Radiotherapy may play an important role in improving prognosis in patients receiving systemic therapy for localized SCPC. In addition, in radiotherapy, it may be better to prioritize irradiation to local lesions with higher doses rather than extensive prophylactic irradiation, given the high frequency of metastasis. Furthermore, the utility of accelerated hyperfractionated irradiation of 45 Gy in 30 fractions, which was demonstrated in localized small-cell lung carcinoma, needs to be evaluated [9].

## Conclusions

In conclusion, we experienced a case of SCPC that developed after hormonal therapy for typical prostate carcinoma, in which stable disease was achieved with chemotherapy and repeated local radiotherapy. Recurrence after primary chemoradiotherapy was seen only within/around the GTV. Repeated local treatment with brachytherapy for local recurrences resulted in relatively long-term disease control. Although further studies are needed, the importance of intensifying local treatment in SCPC is suggested. When radiotherapy and chemotherapy are combined, it may be advisable to limit the irradiation field to the vicinity of the GTV and to increase the irradiation dose as much as possible.
